# Novel Functional Aspect of Antihistamines: The Impact of Bepotastine Besilate on Substance P-Induced Events

**DOI:** 10.1155/2009/853687

**Published:** 2009-06-21

**Authors:** Shun Kitaba, Hiroyuki Murota, Yoko Yahata, Hiroaki Azukizawa, Ichiro Katayama

**Affiliations:** Course of Integrated Medicine, Department of Dermatology, Graduate School of Medicine, Osaka University, 2-2 Yamadaoka, Suita-Shi, Osaka 565-0871, Japan

## Abstract

Besides histamine, substance P (SP) has been demonstrated to play a crucial role in pruritic skin diseases. Although antihistamines are frequently used for pruritic skin diseases, little is known concerning the effect on an SP-induced event such as mast cell degranulation and the upregulation of adhesion molecules or the nitric oxide (NO) synthesis in endothelial cells. Our aim was to study the effect of bepotastine besilate on SP-induced degranulation of rat basophillic leukemia (RBL-2H3) cells and expression of adhesion molecules and NO synthesis in human dermal microvascular endothelial cells (HMVECs). Bepotastine besilate significantly inhibited SP-induced degranulation of RBL-2H3 cells and NO synthesis in HMVECs. Bepotastine besilate significantly inhibited expression of adhesion molecules in HMVESs, while it failed to suppress SP-induced upregulation of the adhesion molecules in HMVECs. Therefore, bepotastine besilate is assumed to act favorably on SP-induced basophil degranulation and NO synthesis in HMVECs.

## 1. Introduction

Although second generation antihistamines generally have been considered to inhibit histamine, recently, their multifunctional aspects, besides the blockade of histamine signaling, have attracted a great deal of attention. Traditionally, the efficacy of type 1 histamine receptor (H1) antihistamines for the treatment of allergic diseases has been primarily attributed to their capacity to reduce the activity of histamine on H1 receptors expressed on endothelial cells, peripheral nerve endings, fibroblasts, and epidermal keratinocytes [1–6]. Therefore, both the first, and the second generation antihistamines are capable of improving the erythema, cutaneous edema, and itch sensation. Meanwhile, it has been reported that a second generation antihistamine inhibited murine contact hypersensitivity more markedly than the first generation antihistamine [7]. Therefore, it is assumed that the action of the second generation antihistamines is unique in the contact hypersensitivity reaction and is not merely derived from its H1 receptor blocking action; however, little is known about the unique effects of second generation antihistamines.

 SP is known to contribute to the formation of an allergic dermatitis *via* mast cell degranulation and vasodilatation. Endothelial cells are also known to possibly be substance *P*-responsive cells, thereby producing nitric oxide (NO) [8, 9], which is known as a vasodilatory factor, and express adhesion molecules including ICAM-1 and *P*-selectin (CD62P) [10, 11] by exposure to SP. From these results, it might be supposed that the inhibition of the SP-elicited response will thus have a favorable effect on allergic diseases. The SP receptor, neurokinin-1 receptor (NK1R), which is widely distributed in peripheral tissues (e.g., endothelial cells, keratinocytes, mast cells, and fibroblasts) [12, 13], has a 7-transmembrane receptor, as well as the H1 receptor. As NK1R antagonists attenuated the histamine-induced behavioral response in mice, the existence of crosstalking between H1R and NK1R has been supposed [14]. Although antihistamines are expected to attenuate the SP-induced events, the first generation antihistamines such as chlorpheniramine failed to inhibit the SP-elicited response [15], second generation antihistamines have not yet been examined.

 In this study, the effect of bepotastine besilate, a second generation antihistamine, against the SP-induced degranulation of RBL-2H3 and the production of nitrite from human microvascular endothelial cells was investigated.

## 2. Materials and Methods

### 2.1. Cell Culture

RBL-2H3, a basophil cell line, was obtained from the Health Science Research Resources Bank (Osaka, Japan). The cells were grown in modified Eagle's medium (Gibco-BRL, Gaithersburg, MD, USA) containing 10% fetal bovine serum (BioWhittaker Inc., Walkersville, MD, USA) and streptomycin at 37°C in a 5% CO_2_ atmosphere. Human microvascular endothelial cells (HMVECs) were obtained from Clonetics^*®*^ Endothelial Cell Systems (Cambrex Bio Science. Walkersville Inc., MD, USA) and were cultured in EBM2 medium (SANKO Junyaku, Ibaragi, Japan) containing 20% fetal bovine serum.

### 2.2. *β*-Hexosaminidase Release Assay

Degranulation was determined by measuring the release of *β*-hexosaminidase. RBL-2H3 cells were grown on 96 well plates (2 × 10^5^ cells/well) and preincubated with 100 *μ*M pyliramine (Sigma) or 100 ng/mL bepotastine besilate (Mitsubishi Tanabe Pharma. Osaka, Japan) for 30 minutes and subsequently stimulated for 24 hours with 1 × 10^−4^ or 1 × 10^−6^ M of SP (Sigma). Following stimulation, the supernatant was incubated with an equal volume of substrate solution (1 mM *P*-nitrophenyl *N*-acetyl-beta-d-glucosamine in 0.05 M citrate buffer, pH 4.5) for 1 hour at 37°C. The enzyme reaction was stopped by the addition of 0.05 M sodium bicarbonate buffer (pH 10.0), and the reaction product was measured at 450 nm using a Model 680 Microplate Reader (BIO-RAD). To determine the total amount of *β*-hexosaminidase released, an equal number of cells were lysed by assay buffer containing 1% (v/v) Triton X-100 prior to incubation with substrate using the same procedure as for the determination of the activity in the supernatant. The data were presented as the ratio (%) of the released *β*-hexosaminidase/*β*-hexosaminidase contained in the lysed cell.

### 2.3. Enzyme-Linked Immunosorbent Assay (ELISA)

The concentrations of soluble ICAM1 (sICAM1) and soluble VCAM1 (sVCAM1) in the culture medium derived from the 24-hour SP-treated HMVECs were measured by using an R&D ELISA kit. Each kit was used following its recommended protocol to measure each value with a Model 680 Microplate Reader (BIO-RAD).

### 2.4. Immunochemical Staining of Adhesion Molecules

The HMVECs were seeded in Lab-Tek^*®*^ chamber slides (NALGENE Labware, Rochester, NY, USA). After 1 day in culture, the cells were preincubated with 100 *μ*M pyliramine or 100 ng/mL bepotastine besilate for 30 minute and subsequently stimulated for 24 hours with 1 × 10^−4^ or 1 × 10^−6^ M SP. The culture cells were rinsed with phosphate buffered saline (PBS) twice and subsequently fixed with 4% paraformaldehyde for 15 minutes. After adequate rinsing with PBS, cells were incubated with murine antihuman CD62P antibody (Chemicon), rabbit antihuman ICAM-1 (Cell Signaling), and Hoechst33342 (molecular probes) for 20 minutes at room temperature. Antimouse IgG FITC-conjugated antibody and antirabbit IgG TRITC-conjugated antibody (DAKO) were used as secondary antibodies. Images of the immunolabeled sections were captured with an immunofluorescence microscope BZ-8000 (Keyence, Osaka, Japan).

### 2.5. Flow Cytometry

The HMVECs were seeded in 6-well collagen-coated dishes. After 1 day in culture, the cells were preincubated with 100 *μ*M pyliramine or 100 ng/mL bepotastine besilate for 30 minutes and subsequently stimulated for 24 hours with 1 × 10^−4^ or 1 × 10^−6^ M SP. The culture cells were rinsed with Phosphate buffered saline (PBS) once and subsequently incubated with collagenase for 20 minutes in 37°C. The cells were harvested and washed with PBS twice. Subsequently, the harvested cells were incubated with FITC-conjugated monoclonal antihuman CD62P (BD Pharmingen) and PE-conjugated monoclonal antihuman CD54 (BD Pharmingen) as well as isotype control monoclonal antibodies (BD Pharmingen) in PBS supplemented with 1% bovine serum albumin and 0.02% NaN3. After being washed, the cells were fixed with 2% formaldehyde containing PBS. The fixed cells were analyzed by FACScan using the CellQuest software program (Becton Dickinson). Data mining was performed using the FLOWJO software program (Tree Star, Inc., Ashland, USA).

### 2.6. Measurement of the Concentration of Endogenous Nitrite

The HMVECs were grown on 24-well plates (5 × 10^5^ cells/well) and preincubated with 100 *μ*M pyliramine or 100 ng/mL bepotastine besilate for 30 minutes and subsequently stimulated for 24 hours with 1 × 10^−6^ M SP. Following stimulation, the supernatants were harvested. The harvested samples were 10 000 MW filtered and 5-fold diluted prior to a Total NO/Nitrite/Nitrate Assay (R&D System). The measurement of the concentration of endogenous nitrite was performed according to the protocols provided by the manufacturer. The results were obtained from the optical density of each sample using a Model 680 Microplate Reader (BIO-RAD) set at 540 nm.

### 2.7. Statistical Analysis

The Prism5 software program (Graph Pad Software, Calif, USA) was used for the statistic analysis. The evaluating of the statistical values was performed by the unpaired *t*-test. A *P* < .05 was considered significant.

## 3. Results

**Figure 1:**
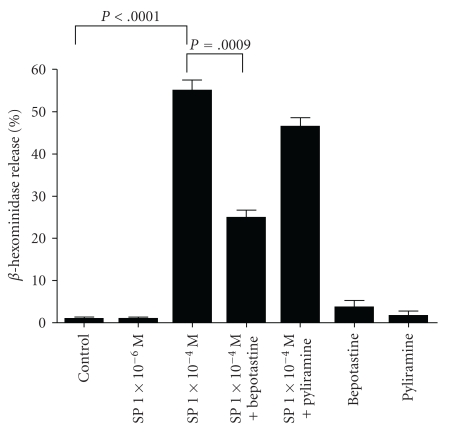
Results of the *β*-hexosaminidase release assay. Control; mock treated. SP: substance *P*. Error bar: standard deviation (SD). *N* = 3–5.

To investigate the effect of antihistamines on the SP-induced basophil degranulation, the concentration of released *β*-hexosaminidase was measured (Figure 1). As expected, 1 × 10^−4^ M of SP-induced the release of *β*-hexosaminidase, while 1 × 10^−6^ M of SP failed to induce the release. Interestingly, bepotastine besilate significantly suppressed the SP-mediated release of *β*-hexosaminidase (*P* = .0009, unpaired *t*-test, 1 × 10^−4^ M of SP versus 1 × 10^−4^ M of SP + bepotastine), while pyliramine failed to suppress. This result suggests that bepotastine besilate potentially affects substance *P* signaling.

**Figure 2:**
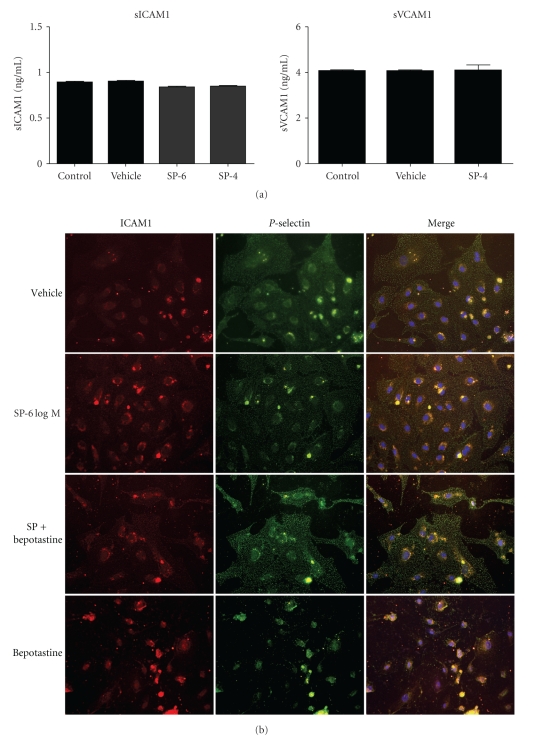
Effects of SP on the expression level of adhesion molecules on HMVEC. (a) The concentration of sICAM1 and sVCAM1 in the conditioned medium derived from SP-treated HMVEC. *N* = 3. Error bar: SD. (b) Immunolabeling of ICAM1 and *P*-selectin on HMVEC with various treatments. Magnification, ×200.

**Figure 3:**
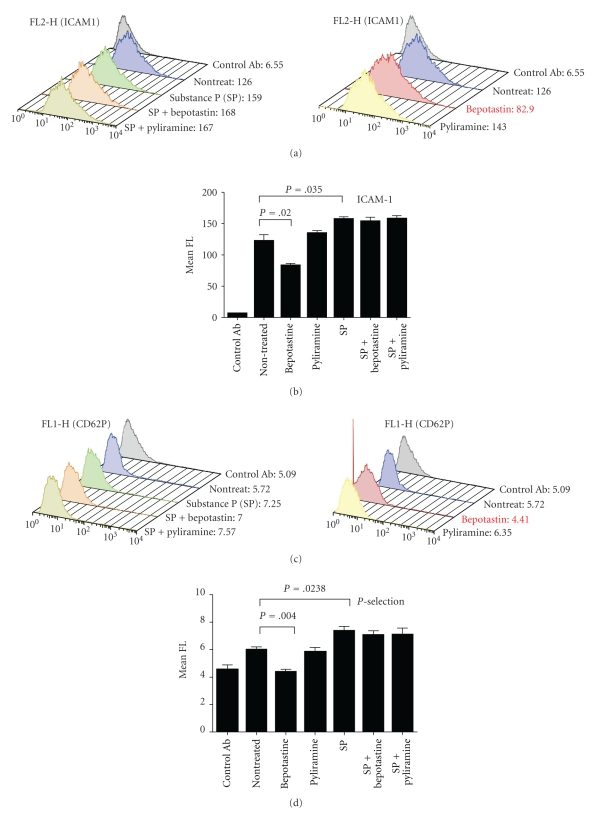
Cell surface expression level of ICAM1 and *P*-selectin on HMVEC with various treatments. (a) Representative data of an FACS analysis for ICAM1. The *X* axis indicates the fluorescence intensity. The *Y* axis represents the cell count number. The heading includes the data of mean fluorescence. (b) The graph shows the mean fluorescence intensity of immunolabeled ICAM1. Mean FL: mean fluorescence. Error bar: SD. *N* = 3. (c) Representative data of an FACS analysis for *P*-selectin. The *X* axis indicates the fluorescence intensity. The *Y* axis represents the cell count number. The heading includes the data of mean fluorescence. (d) The graph shows the mean fluorescence intensity of immunolabeled *P*-selectin. Mean FL: mean fluorescence. Error bar: SD. *N* = 3.

Next, to verify the response of the HMVECs against SP, the HMVECs were treated with 1 × 10^−6^ and 1 × 10^−4^ M of SP or vehicle for 24 hours, and the medium concentration of sICAM1 and sVCAM1 were measured (Figure 2(a)). Unexpectedly, no significant differences were observed between the SP-treated and untreated groups. To verify the localization and expression level of ICAM1 and *P*-selectin in the HMVECs, immunochemical staining was performed (Figure 2(b)). However, there are no significant changes between the SP-treated and untreated groups. Interestingly, when the HMVECs were treated with bepotastine besilate alone, the cell surface expression level of ICAM-1 or *P*-selectin had a tendency to decrease (Figure 2(b)). To verify the above results, an FACS analysis for ICAM-1 and *P*-selectin was performed (Figure 3). The cell surface expression level of ICAM-1 and *P*-selectin slightly, but significantly, increased on the SP-treated HMVECs. Although, both bepotastine besilate and pyliramine did not affect the SP-induced upregulation of ICAM-1 and *P*-selectin, a significant decrease of the expression level of these adhesion molecules on the HMVECs treated with bepotastine besilate alone was reproduced by this assay.

**Figure 4:**
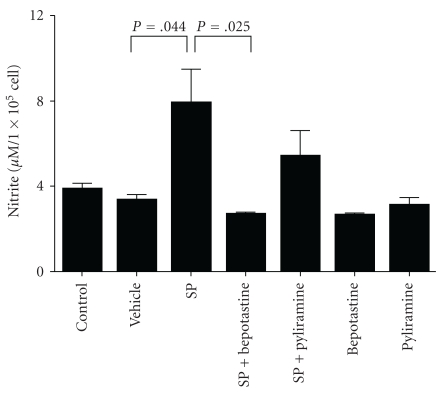
The concentration of nitrite in conditioned medium derived from HMVEC cultured with substance *P* and antihistamines. Error bars: SD. *N* = 5–6.

Next, the concentration of synthesized nitrite in the culture medium derived from the SP-treated HMVECs, with or without antihistamine, was measured. The SP (1 × 10^−6^ M) treated HMVECs derived from the conditioned medium contained a significantly high amount of nitrite in comparison to the control and vehicle treated medium. Interestingly, the SP-induced synthesis of nitrite was significantly inhibited by bepotastine besilate, while that was not inhibited by pyliramine-treatment. This data suggests that bepotasitine besilate potentially inhibits the SP-induced NO synthesis of endothelial cells (Figure 4).

## 4. Discussion

This report may identify a novel functional aspect of the second generation antihistamines against the effect of SP on basophils and endothelial cells. It has been demonstrated that mast cells populated in the chronic allergic skin diseases such as atopic dermatitis, and prurigo nodularis [5]. Although, histamine, which is released from the mast cell, is essential for maintaining homeostasis in living organisms [16, 17], an excessive response to histamine has been shown to play an important role in the pathogenesis of chronic allergic diseases, including atopic dermatitis [5]. From these facts, the inhibition of excessive mast cell degranulation might be a promising treatment of allergic skin diseases. 

 The mast cells of the human skin, but not those of other tissues, are sensitive to stimulation by substance *P*, compound 48/80, and other basic nonimmunological stimuli [18]. The mechanism of mediator secretion induced by these agents is distinct from that induced by IgE-dependent stimulation [18]. As shown in Figure 1, bepotastine besilate, but not pyliramine, inhibited the SP-induced degranulation of RBL-2H3. From this result, it is supposed that bepotastine besilate might have an inhibitory action on the distinct pathway of SP-induced degranulation besides the H1-antagonistic action. Moreover, the FACS analysis caused the unexpected result of a downregulation of the adhesion molecules on the bepotastine besilate-treated HMVECs (Figure 3). These advantages of bepotastine besilate will provide good benefits for the treatment of various kinds of allergic skin diseases. However, there are strong doubts about whether bepotastine besilate acts directly on SP-mediated signaling or not, because bepotastine besilate has failed to inhibit the SP-induced upregulation of the adhesion molecules on the HMVECs (Figures 2 and 3). Although, it is easy to assume that the effect of bepotastine besilate might depend on the cell type, further examinations are still needed to resolve this question. 

 This report also showed the remarkable results of the effect of bepotastine besilate on nitrite synthesis from the SP-treated HMVECs. NO is a gaseous lipophilic free radical cellular messenger that has pleiotropic actions including regulation of blood pressure and vascular tone, inhibition of platelet aggregation and leukocyte adhesion, and prevention smooth muscle cell proliferation. NO is also known to contribute to the formulation of various kinds of skin diseases, such as urticaria [19], atopic dermatitis (AD) [20], psoriasis [21], pemphigus [20], and systemic lupus erythematosis [22]. Above all, it is striking that the serum nitrate levels of the patients with AD significantly increased in comparison to the nonatopic controls and were also correlated with the disease severity [20]. Because, there are no reports describing any correlation between AD and decreased blood pressure and no experience of examining AD patients who suffer from decreased blood pressure in our daily clinical practice, it is therefore assumed that an increased serum concentration of NO in the AD patients does not affect the patient's physiological condition. It does, however, contribute to the exacerbation of skin eruptions. In this study, bepotastine besilate was found to significantly inhibit the SP-induced nitrite synthesis in the HMVECs, while pyliramine failed to inhibit it (Figure 4). It should therefore be considered that no side effects of hypotension have, thus far, been reported in patients treated with bepotastine besilate, thus suggesting it to be potentially useful as a safe and promising antihistamine for patients with NO-related skin diseases. 

 In fact, the above-mentioned possible clinical effects of bepotastine besilate are still hypothetical. It is therefore of the utmost importance that more clinical data be generated regarding these parameters in bepotastine besilate-treated patients.
